# Role of Extracorporeal Membrane Oxygenation in Adults and Children With Refractory Septic Shock: A Systematic Review and Meta-Analysis

**DOI:** 10.3389/fped.2021.791781

**Published:** 2022-01-21

**Authors:** Yufan Yang, Zhenghui Xiao, Jiaotian Huang, Ling Gong, Xiulan Lu

**Affiliations:** Department of Intensive Care Unit of Hunan Children's Hospital, Changsha, China

**Keywords:** children, refractory septic shock, extracorporeal membrane oxygenation, adults, survival

## Abstract

**Background:**

The benefits of extracorporeal membrane oxygenation in patients with refractory septic shock remain controversial. Current guidelines on the management of refractory septic shock recommend the consideration of extracorporeal membrane oxygenation as a salvage therapy. The difference between adults and children with septic refractory shock treated with extracorporeal membrane oxygenation has not been previously analyzed. We aimed to review peer-reviewed publications on the role of extracorporeal membrane oxygenation in adults and children with refractory septic shock.

**Methods:**

Studies reporting on mortality in both adults and children with refractory septic shock supported with extracorporeal membrane oxygenation published in PubMed, Cochrane Library, and Embase databases were included in the meta-analysis. Study eligibility was independently assessed by two authors, and disagreements were resolved by a third author. The outcome measure was survival at discharge. Subgroup analysis included the adult and pediatric groups.

**Results:**

Of the 293 articles screened, 14 original articles were identified for systematic review and meta-analysis. The cumulative estimate of survival (14 studies, 535 patients) in the cohort was 39% (95% confidence interval [CI]: 27–51%). During the subgroup analysis, the cumulative estimate of survival at discharge in the adult group (6 studies, 276 patients) in the cohort was 18% (95% CI: 10–27%), and that in the pediatric group (8 studies, 259 patients) was 53% (95% CI: 47–59%).

**Conclusions:**

The survival rate of adults with refractory septic shock requiring extracorporeal membrane oxygenation was 18%, and children with refractory septic shock requiring extracorporeal membrane oxygenation had a higher survival rate (53%) than adults.

## Introduction

In recent years, the use of extracorporeal membrane oxygenation (ECMO) in both adult and pediatric patients has increased significantly (1). Refractory septic shock is a clinical condition caused by a dysregulated host response to infection and is characterized by the presence of refractory hypotension and a high serum lactate level (2). The American College of Critical Care Medicine has suggested that ECMO is a viable therapy for refractory septic shock that is unresponsive to all other conservative treatments (3). However, although successful use of ECMO in adults with refractory septic shock has been reported in a few cases (4–6), reports of ECMO in adults and children with refractory septic shock remain limited. In addition, no study has explored the difference in the use of ECMO in refractory septic shock between adults and children. As a result, the outcome benefits of ECMO in refractory septic shock remain controversial. We aimed to systematically review the literature to examine the survival rates of adult and pediatric patients with refractory septic shock requiring ECMO and to discuss the differences between them.

## Methods

This study adhered to the ethical guidelines of the Declaration of Helsinki following the Preferred Reporting Items for Systematic Review and Meta-Analysis statement (7). Publications were reviewed for quality using the Joanna Briggs Institute (JBI) checklist for prevalence studies (8) and the Grading of Recommendations Assessment Development and Evaluation (GRADE) system to determine the overall rating confidence in the body of evidence (9).

### Study Selection

Inclusion criteria: (1) study design: the study was original research from prospective or retrospective studies; (2) participants: adults and children; (3) exposure: refractory septic shock treated with ECMO; (4) comparator: survival at discharge; and (5) outcome: death or survival. Exclusion criteria: the review, commentaries, opinions, guidelines, pathological studies. Two investigators independently searched PubMed, Embase, and Cochrane Library databases for studies that enrolled patients with refractory septic shock treated with ECMO, which were published before July 9, 2021 and restricted to English. The search phrases for the three databases included Boolean terms “AND” and “OR” with the following keywords in various possible combinations: “ECMO,” “extracorporeal membrane oxygenation,” “Infant,” “newborn,” “neonatal,” “pediatric,” “adults,” and “refractory septic shock” (details of the search strategy are included in the Supplementary Material). Two authors screened titles, abstracts, or full texts and determined their eligibility. In addition, a manual search of all relevant studies and their citation lists was performed to identify additional articles for inclusion. And no restrictions were placed on study type (prospective or retrospective) because we have used the Joanna Briggs Institute Checklist to assess all the studies and put it in the Supplementary Data.

### Study Analysis

Meta-analysis was performed using R software. Data including study design, outcomes, patient characteristics, and interventions were extracted independently. Survival to discharge was the outcome measure in our meta-analysis. Subgroup analysis was also performed to analyze the differences between the adult and pediatric groups. Briefly, for the meta-analysis of proportions, the exact confidence interval (CI) for each proportion was computed using the Clopper-Pearson method (10). *I*^2^-tests were performed to assess the heterogeneity of the summary rates. Statistical heterogeneity between studies was identified using *I*^2^ statistics, where *I*^2^ ≤ 40%, between 30 and 60%, between 50 and 75%, and ≥75% indicated low, moderate, substantial, and considerable heterogeneity, respectively. *P*-values for *I*^2^ statistics were derived from the chi-square distribution of Cochran's *Q*-test.

## Results

### Study Characteristics

**Figure 1:**
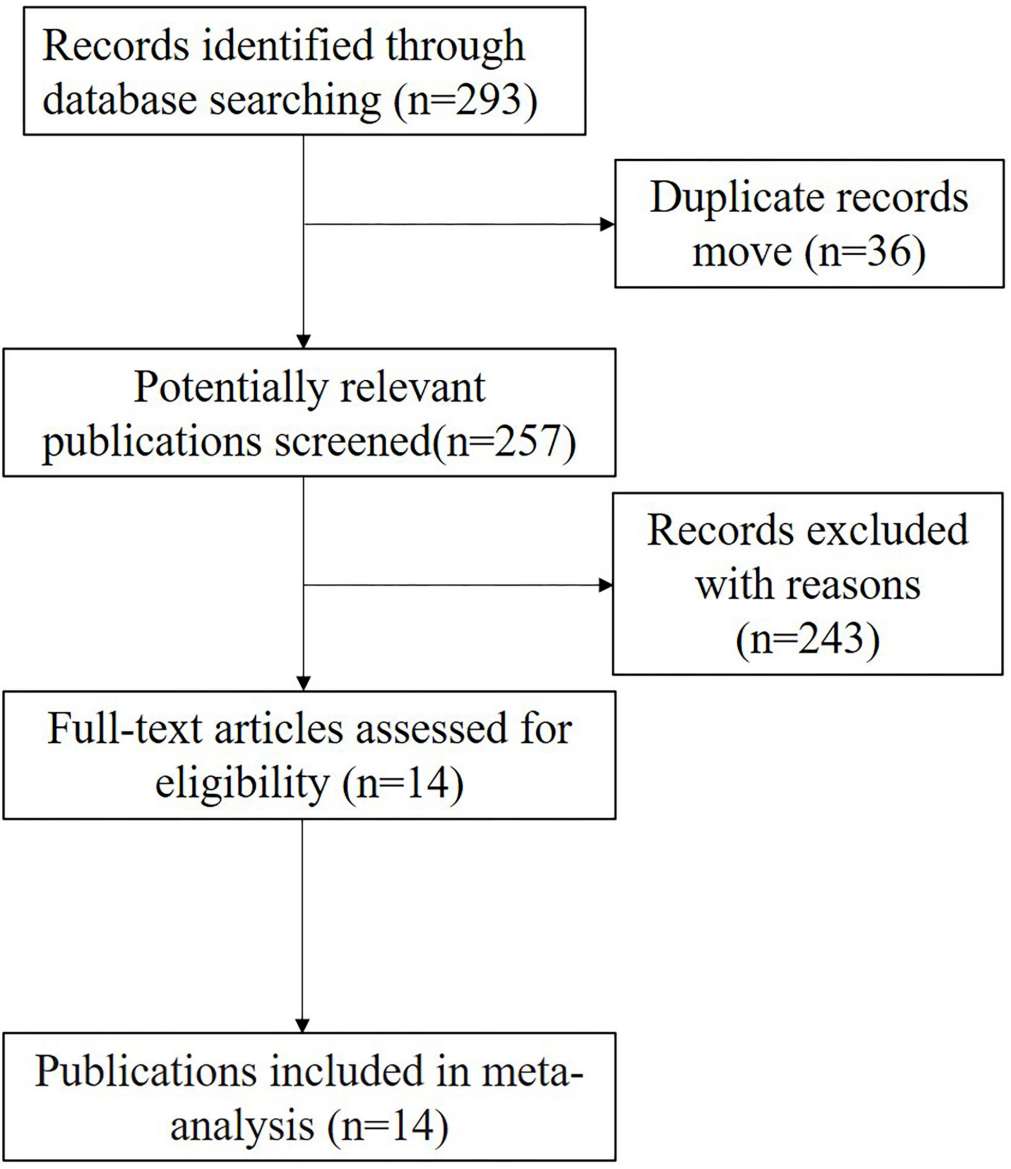
Flow chart showing the process of study selection and numbers of studies included. A total of 293 studies were found in PubMed, Embase, and the Cochrane Library databases. And there were 14 studies included in this meta-analysis.

The results of the study selection process are presented in Figure 1. A total of 293 records were found in PubMed, Embase, and the Cochrane Library databases. And we excluded 36 records and 243 irrelevant records (review, commentaries, opinions, guidelines, pathological) in this meta-analysis. After excluding duplicates, irrelevant studies, and studies without corresponding data, we eventually identified 14 studies that met the inclusion criteria.

### Clinical Outcomes

**Figure 2:**
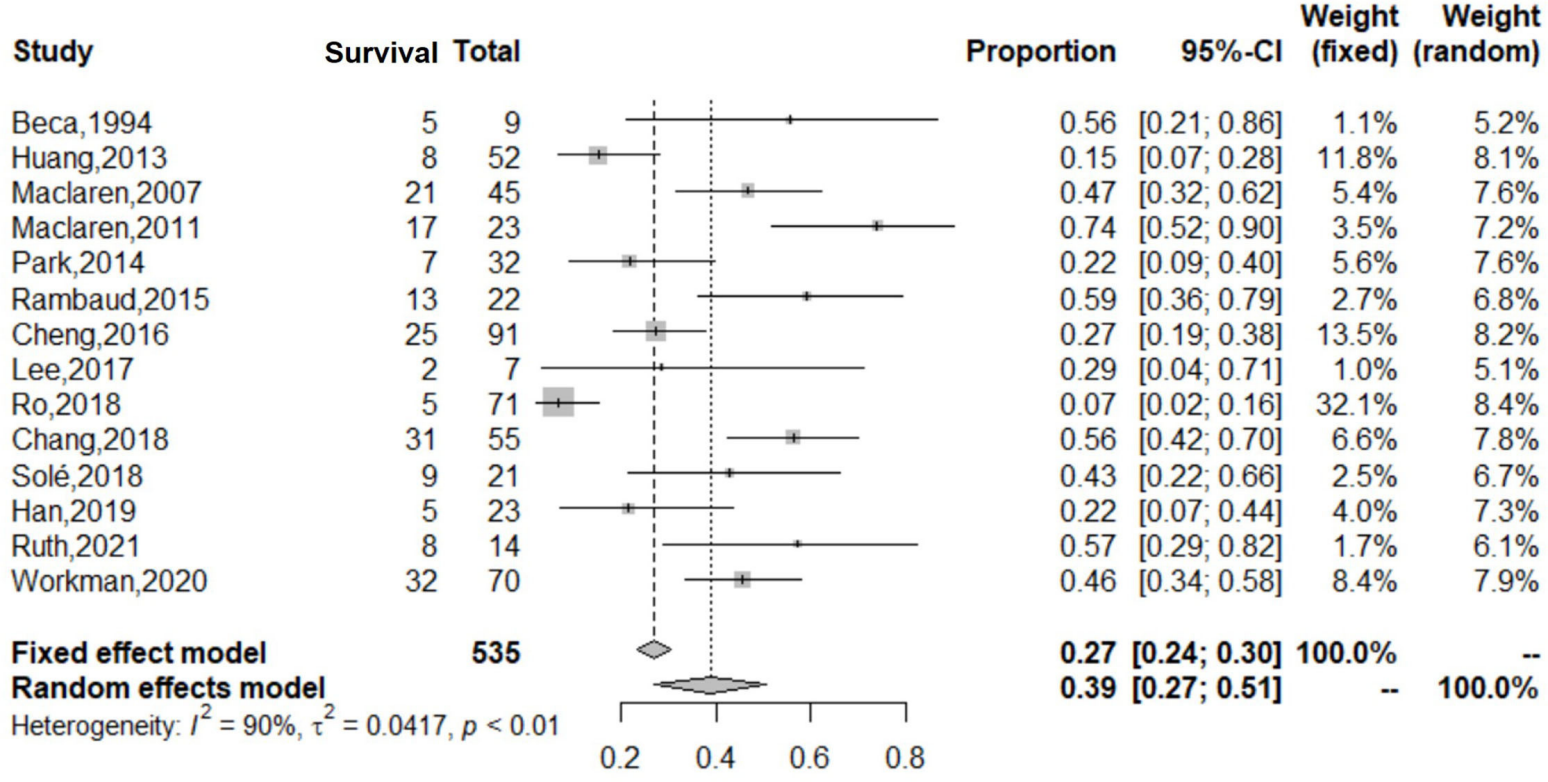
Forest plot of studies reporting on use of ECMO in both adults and children with refractory septic shock. The cumulative estimate of survival (14 studies, 535 patients) in the cohort was 39% (95% CI: 27–51%). CI, confidence interval.

**Figure 3:**
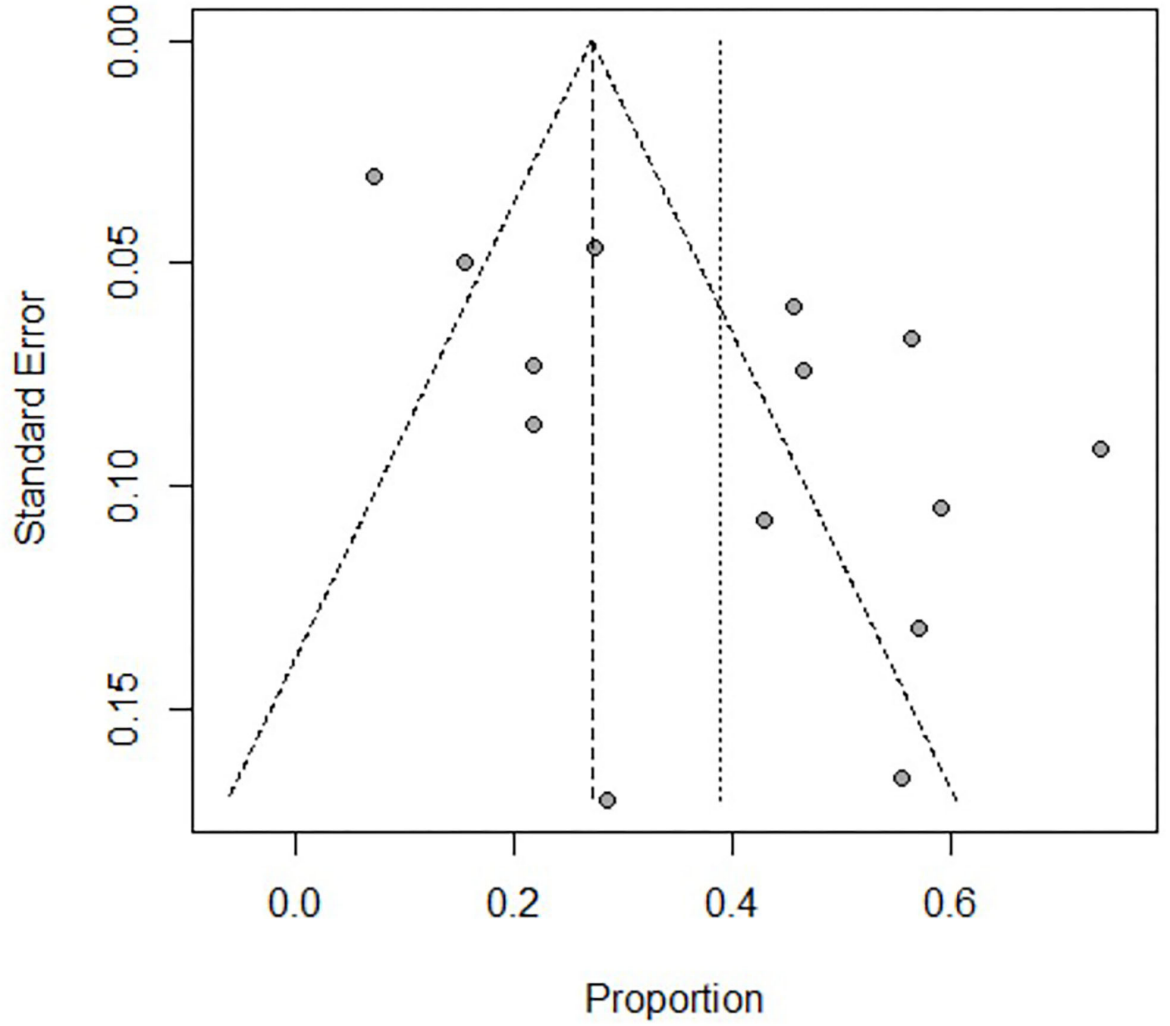
Assessment of the funnel plot of both adults and children with refractory septic shock treated with ECMO. It shows that the heterogeneity is great before dividing into subgroups.

Of the 293 articles screened, 14 were identified for systematic review and meta-analysis. The cumulative estimate of survival (14 studies, 535 patients) in the cohort was 39% (95% CI: 27–51%) (Figure 2). An assessment of the funnel plots of all the patients is shown in Figure 3. The 14 studies included in the systemic review and meta-analysis that reported on children with refractory septic shock requiring ECMO are shown in Table 1 (11–24). And the details of the cohort studies are summarized in Table 2. Furthermore, the compilation of adverse events associated with the use of ECMO are provided in Table 3.

**Table 1:** List of 13 articles included in systemic review and meta-analysis that reported on patients with refractory septic shock needing ECMO.

**Author**	**Period**	**Sample size**	**Survival**	**Type of ECMO**	**Children or adults**
Beca and Butt (11)	1989–1991	9	5	VA	Children
Huang et al. (12)	2005–2010	52	8	VA	Adults
MacLaren et al. (13)	1988–2006	45	21	VA	Children
MacLaren et al. (14)	2000–2009	23	17	Central and VA	Children
Park et al. (15)	2005–2013	32	7	VA	Adults
Rambaud et al. (16)	2004–2013	22	13	VA	Children and neonatals
Cheng et al. (17)	2001–2011	91	25	VA	Adults
Lee et al. (18)	2005–2012	7	2	VA	Adults
Ro et al. (19)	2005–2012	71	5	VA	Adults
Chang et al. (20)	2008–2015	55	31	VA	Children
Solé et al. (21)	2001–2017	21	9	VA	Children and neonatals
Han et al. (22)	2007–2017	23	5	VA	Adults
Ruth et al. (23)	2011–2018	14	8	Central and VA	Children
Workman et al. (24)	2005–2011	70	32	VA	Children and neonatals

**Table 2:** Details of the cohort studies.

**Author**	**Age (years, months or days)**	**Male**	**Female**	**Blood lactate before ECMO (mmol/L)**	**Duration of ECMO (hours)**
Beca and Butt (11)	12y (0.2–15)	4	5	/	137 (57–231)
Huang et al. (12)	56.8y (42.7–63.6)	39	13	Survivors:5.3 Non-survivors:8.8	43.3 (10.2–157.5)
MacLaren et al. (13)	2.5y (0.4–9)	28	17	8.1 (5.1–12.3)	84 (32–135)
MacLaren et al. (14)	6y (2.8–12.3)	13	10	7.8 (4.1–9.7)	93 (43–119)
Park et al. (15)	55y (44–63)	21	11	8.9 (5.8–14.6)	84 (43.7–115.2)
Rambaud et al. (16)	30 m (1–113)	14	8	Neonatals:7.94 ± 4.92 Pediatrics: 5.2 ± 3.5	Neonatals:178 (24–408) Pediatrics: 141.6 (72–240)
Cheng et al. (17)	Unknown	/	/	7.2 ± 5.3	Unknown
Lee et al. (18)	48y (19–67)	6	2	12.29 (6.7–20.6)	96(24–312)
Ro et al. (19)	57.5y (48–65)	40	31	Survivors:5.8 (4.3–5.9) Non-survivors: 11.6 (7.5–15.0)	Unknown
Chang et al. (20)	7.2y ± 6.19	29	26	/	216 (0–2472)
Solé et al. (21)	Pediatrics: 3.3y (0.7–4.7) Neonatals: 1d (1–5)	14	7	13.3 (5.6–17.8)	84 (21–120)
Han et al. (22)	Survival: 45y (20–62) Death: 54y (48–61)	14	9	Survival: 4.4 (2.2–7.4) Death: 6.8 (5.5–8.9)	Survival: 146 (125.5–167.5) Death: 159 (142.5–205.3)
Ruth et al. (23)	104.5 m (17–166.75)	4	10	Survivors: 4.5 (3.8–11.7) Non-survivors: 4.8(2.0–12.4)	147.1 (91.9–178.6)
Workman et al. (24)	unknown	32	38	6.9 (3.9–10.1)	132 (67.2–225.6)

**Table 3:** Compilation of adverse events associated with the use of ECMO.

**Author**	**Sample size**	**Bleeding complications**	**Blood clots in the circuit**	**Limb ischaemia**	**Stroke**	**Neurological sequelae**
Beca and Butt (11)	9	4	1	/	/	/
Huang et al. (12)	52	4	1	/	/	/
MacLaren et al. (13)	45	/	7	/	/	/
MacLaren et al. (14)	23	8	11	/	/	/
Park et al. (15)	32	3	/	5	/	/
Rambaud et al. (16)	22	1	9	/	2	/
Cheng et al. (17)	91	/	/	/	/	/
Lee et al. (18)	7	/	/	/	/	/
Ro et al. (19)	71	/	/	1	/	/
Chang et al. (20)	55	/	/	/	/	6
Solé et al. (21)	21	1	10	/	/	/
Han et al. (22)	23	/	/	/	/	/
Ruth et al. (23)	14	10	/	/	/	/
Workman et al. (24)	70	51	/	/	/	/

### Subgroup Analysis

**Figure 4:**
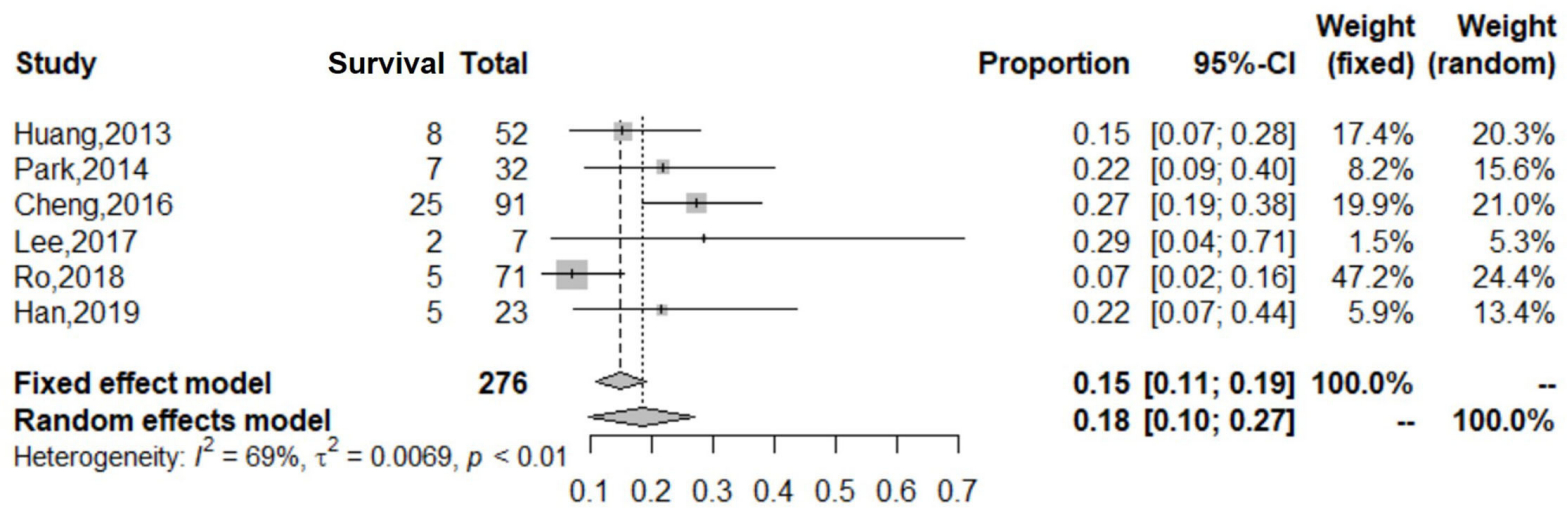
Forest plot of studies reporting on use of ECMO in adults with refractory septic shock. The cumulative estimate of survival in the adult group (6 studies, 276 patients) in the cohort was 18% (95% CI: 10–27%). CI, confidence interval.

**Figure 5:**
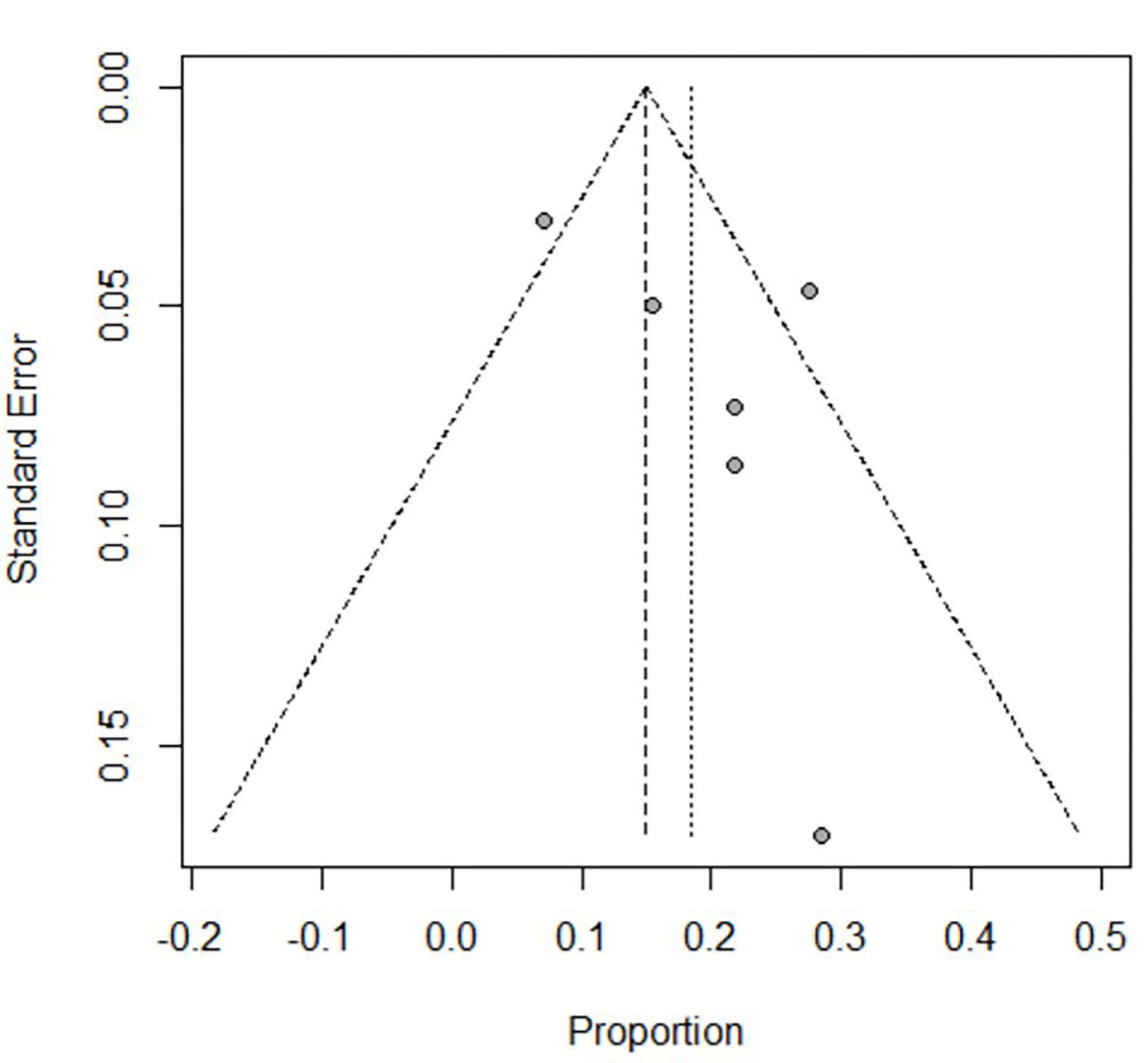
Assessment of the funnel plot of adults with refractory septic shock treated with ECMO. The heterogeneity in the funnel plots of adults was lower than after dividing into subgroups.

**Figure 6:**
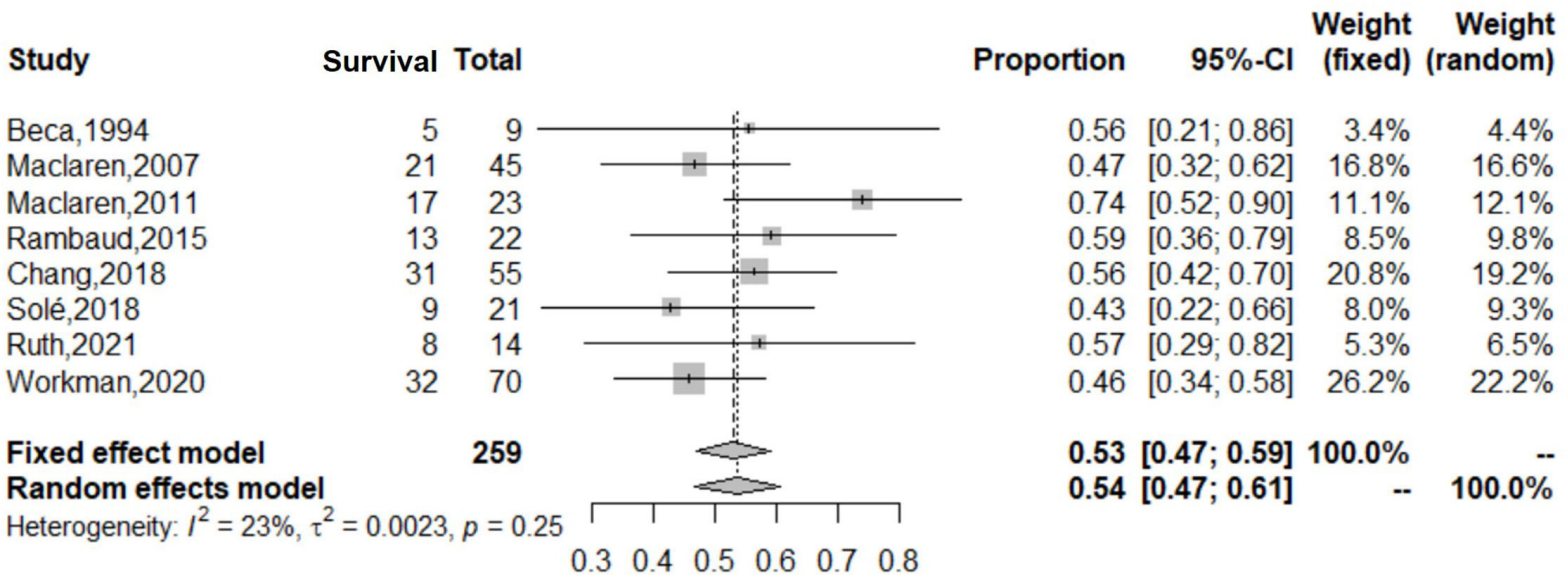
Forest plot of studies reporting on use of ECMO in children with refractory septic shock. The cumulative estimate of survival in the pediatric group (8 studies, 259 patients) in the cohort was 53% (95% CI: 47–59%). CI, confidence interval.

**Figure 7:**
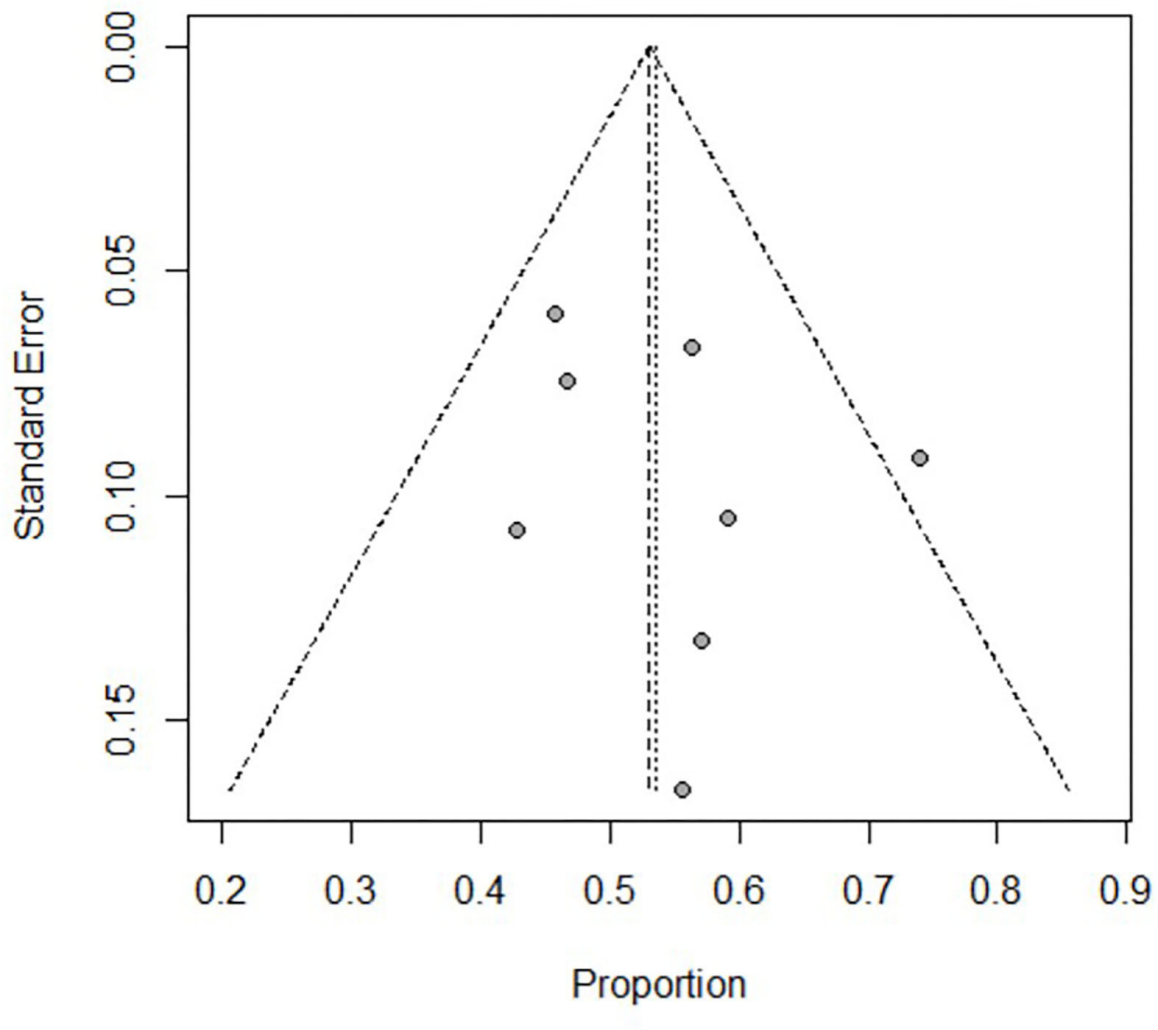
Assessment of the funnel plot of children with refractory septic shock treated with ECMO. The heterogeneity in the funnel plots of children was lower than after dividing into subgroups.

During the subgroup analysis, the cumulative estimate of survival in the adult group (6 studies, 276 patients) in the cohort was 18% (95% CI: 10–27%) (Figure 4), and an assessment of the funnel plot of adults is shown in Figure 5. The cumulative estimate of survival in the pediatric group (8 studies, 259 patients) in the cohort was 53% (95% CI: 47–59%) (Figure 6), and the assessment of the funnel plot of children is shown in Figure 7.

### Quality Assessment

There was no evidence of publication bias, and the methodological quality of all the studies included in our analysis scored high using the JBI critical appraisal tool (Supplementary Table 1). The GRADE analysis demonstrated moderate to high certainty in the evidence presented in this paper.

## Discussion

The application of ECMO in patients with refractory septic shock has increased over the past decade, with variable survival rates (25, 26). Our meta-analysis of observational studies showed that the overall survival rate of patients (adults and children) with refractory septic shock treated with ECMO was 39%, while in the subgroup analysis, the cumulative estimate of survival in the adult group (6 studies, 276 patients) in the cohort was 18% (95% CI: 11–19%), and that in the pediatric group (8 studies, 259 patients) in the cohort was 53% (95% CI: 47–59%). The benefits of extracorporeal support include improved global oxygen delivery, reduced intrathoracic pressures from reduced mechanical ventilatory requirements, improved carbon dioxide clearance and acid-base management, and improved myocardial performance (27).

### Subgroup Analysis

There was great heterogeneity in the forest and funnels plots of all patients (Figures 2, 3). Consequently, we selected the random-effects model to assess the overall survival rate of patients treated with ECMO, which was 39%. We divided the patients into two subgroups: adults, and children and neonates.

The adult group with refractory septic shock treated with ECMO had significantly lower survival rates (18%) than pediatric group. In addition, the heterogeneity in the forest and funnel plots of adults was lower than that of the overall patients (Figures 4, 5). The pediatric and neonate group with refractory septic shock treated with ECMO had significantly higher survival rates than the adult groups. In addition, the heterogeneity in the forest and funnel plots of pediatrics was lower than that of the overall patients (Figures 6, 7). This indicates that our subgroup analysis is of great significance in reducing heterogeneity.

Huang et al. have concluded that for patients with refractory septic shock treated with ECMO, the older the adult, the worse the prognosis. All 20 adults aged over 60 years died despite the use of ECMO, which indicates that the outcomes of these patients remain unsatisfactory. There are three possible explanations for this phenomenon: First, refractory septic shock has a variety of hemodynamic presentations. Left ventricular dysfunction with reduced cardiac output is commonly seen in infants and children; however, distributive shock, a hyperdynamic state with high cardiac output, usually manifests in adults (28). ECMO is used primarily for cardiac or cardiopulmonary support and is intuitively more beneficial for patients with ventricular dysfunction than for those with profound vasodilation. Thus, the performance of ECMO is better in pediatric than adult refractory septic shock. Second, among adults, older patients have more comorbid diseases (such as diabetes and hypertension), which are more difficult to recover from after ECMO for life support. Thus, the adult survival rates declined at discharge. According to our experience, although some children with refractory septic shock who need ECMO in PICU also have chronic comorbidity such as type 1 diabetes, congenital heart disease, chronic lung disease, the incidence rate is lower than that of adults, and the exposed time to chronic diseases is shorter than adults. Third, cardiovascular diseases increase dramatically with age in human. While it is clear that advanced age allows more time for individuals to be exposed to risk factors in general, there is strong evidence that age itself is a major independent risk factor for death (29). These three points may be the cause of the significant differences between the adult and pediatric groups.

### Shock-to-Extracorporeal Membrane Oxygenation Interval

Han et al. (22) have reported that the shock-to-ECMO interval for adults at 12, 18, and 24 h during ECMO between the survival and death groups was significantly different. They found that shock-to-ECMO interval before ECMO placement in the survival group were significantly lower than those in the death group (23.5 vs. 42.2 h, *P* = 0.037). Cheng et al. (17) have found that better outcomes were associated with in ECMO patients with door-to-ECMO times of 96 h or less. Solé et al. (21) have reported 21 refractory septic shock patients treated with ECMO, 9 were pediatric and 12 were newborns. And they were diagnosed with septic shock for a median duration of 29.5 h before ECMO was started (IQR, 20–46), with significant differences between the survival and non-survival groups (*P* = 0.009). These three studies indicate that the shorter the time from refractory septic shock to ECMO, the higher the survival rate at discharge.

### Study Limitations

First, this study lacked long-term survival rate for patients with refractory septic shock treated with ECMO; however, ECMO is a supportive method, not a treatment. The most important points in patient management are the control of the focus of infection and the early initiation of adequate antibiotics. Second, the number of the cases in some studies included in this meta-analysis are small. Thus, further multicenter studies conducted with larger sample sizes are needed to confirm our findings.

## Conclusions

The survival rate of adults with refractory septic shock requiring ECMO was 18%, and children with refractory septic shock requiring ECMO had a higher survival rate (53%) than adults.

## Data Availability Statement

The original contributions presented in the study are included in the article/Supplementary Material, further inquiries can be directed to the corresponding author/s.

## Author Contributions

XL: conception and design. ZX: administrative support. YY and JH: provision of study materials or patients. YY and LG: collection and assembly of data and data analysis and interpretation. All authors manuscript writing and final approval of manuscript.

## Funding

This work was funded by the Hunan Provincial Science and Technology Department Project (No. 2018SK2135, 2020SK1014-3), the Hunan Provincial Key Laboratory of Emergency Medicine for Children (No. 2018TP1028), and the Hunan Provincial Key Laboratory of Metabolomics in Critical Care Medicine (No. 2017TP1034). The funders had no role in study design, data collection and analysis, decision to publish, or preparation of the manuscript.

## Conflict of Interest

The authors declare that the research was conducted in the absence of any commercial or financial relationships that could be construed as a potential conflict of interest.

## Publisher's Note

All claims expressed in this article are solely those of the authors and do not necessarily represent those of their affiliated organizations, or those of the publisher, the editors and the reviewers. Any product that may be evaluated in this article, or claim that may be made by its manufacturer, is not guaranteed or endorsed by the publisher.

## Abbreviations

ECMO: Extracorporeal membrane oxygenation
JBI: Joanna Briggs Institute
GRADE: the Grading of Recommendations Assessment Development and Evaluation
CI: Confidence interval.
