# Supplementary material for: Role of Extracorporeal Membrane Oxygenation in Adults and Children With Refractory Septic Shock: A Systematic Review and Meta-Analysis
Source: Front Pediatr. 2022 Jan 21;9:791781. doi: 10.3389/fped.2021.791781 (PMC8814621; doi:10.3389/fped.2021.791781)
Supplement: Supplementary file 1 [file Data_Sheet_1.docx]

**Supplement methods**

Search strategy

Pubmed database

#1 "Extracorporeal Membrane Oxygenation"[Mesh] OR ECMO[Title/Abstract] OR "Extracorporeal Membrane Oxygenation"[Title/Abstract] OR “extracorporeal life support”[Title/Abstract] OR “extracorporeal membrane oxygenator support”[Title/Abstract]

#2 “Infant”[Title/Abstract] OR “newborn”[Title/Abstract] OR “child”[Title/Abstract] OR “adult”[Title/Abstract] OR “pediatric”[Title/Abstract] OR “patient”[Title/abstract] OR “neonate”[Title/abstract] OR “Infant”[Mesh] OR “newborn”[Mesh] OR “children”[Mesh] OR “adult”[Mesh] OR “pediatric”[Mesh] OR “patient”[Mesh] OR “neonate”[Mesh]

#3 "refractory septic shock"[Mesh] OR “refractory septic shock”[Title/Abstract]

#1 and #2 and #3

Embase database

#1. 'child'/exp OR child OR 'adult'/exp OR adult OR 'patient'/exp OR patient OR infants OR 'newborn'/exp OR newborn OR neonate

#2. 'refractory septic shock' OR (refractory AND septic AND ('shock'/exp OR shock))

#3. ECMO OR 'extracorporeal membrane oxygenation'/exp OR 'extracorporeal membrane oxygenation' OR (extracorporeal AND ('membrane'/exp OR membrane) AND ('oxygenation'/exp OR oxygenation)) OR 'extracorporeal life support'/exp OR 'extracorporeal life support' OR (extracorporeal AND ('life'/exp OR life) AND support) OR 'extracorporeal membrane oxygenator support' OR (extracorporeal AND ('membrane'/exp OR membrane) AND ('oxygenator'/exp OR oxygenator) AND support) OR 'extracorporeal oxygenation'/exp OR 'extracorporeal oxygenation' OR (extracorporeal AND ('oxygenation'/exp OR oxygenation))

#1 AND #2 AND #3

Cochrane library

#1 "Extracorporeal Membrane Oxygenation" OR “ECMO” OR “extracorporeal life support” OR “extracorporeal membrane oxygenator support”

#2 "refractory septic shock"

#3 “Infant” OR “newborn” OR “child” OR “adult” OR “pediatric” OR “patient” OR “neonate”

#1 AND #2 AND #3

Supplementary Table 1: Outcomes of the Joanna Briggs Institute Checklist for prevalence studies.

| Author | Year | Question number | | | | | | | | | | Overall score |
| --- | --- | --- | --- | --- | --- | --- | --- | --- | --- | --- | --- | --- |
|  |  | 1 | 2 | 3 | 4 | 5 | 6 | 7 | 8 | 9 | 10 | 10 |
| John Beca | 1994 | ✓ | ✓ | ✓ |  | ✓ | ✓ | ✓ | ✓ | ✓ | ✓ | 9 |
| Chun-Ta Huang | 2013 | ✓ | ✓ | ✓ | ✓ | ✓ |  | ✓ | ✓ | ✓ | ✓ | 9 |
| Graeme MacLaren | 2007 | ✓ | ✓ | ✓ | ✓ | ✓ | ✓ | ✓ | ✓ | ✓ | ✓ | 10 |
| Graeme MacLaren | 2011 | ✓ | ✓ | ✓ | ✓ | ✓ | ✓ | ✓ | ✓ | ✓ | ✓ | 10 |
| Taek Kyu Park | 2014 | ✓ | ✓ | ✓ | ✓ | ✓ | ✓ | ✓ | ✓ | ✓ | ✓ | 10 |
| Jerome Rambaud | 2015 | ✓ | ✓ | ✓ | ✓ | ✓ |  | ✓ | ✓ | ✓ | ✓ | 9 |
| Aristine Cheng | 2016 | ✓ | ✓ | ✓ | ✓ | ✓ | ✓ | ✓ | ✓ | ✓ | ✓ | 10 |
| [Kyo Won Lee](https://pubmed.ncbi.nlm.nih.gov/?size=50&term=Lee+KW&cauthor_id=28932731) | 2017 | ✓ | ✓ | ✓ | ✓ | ✓ | ✓ | ✓ | ✓ | ✓ | ✓ | 10 |
| Sun Kyun Ro | 2018 | ✓ | ✓ | ✓ | ✓ | ✓ | ✓ | ✓ | ✓ | ✓ | ✓ | 10 |
| Tu-Hsuan Chang | 2018 | ✓ | ✓ | ✓ | ✓ | ✓ |  | ✓ | ✓ | ✓ | ✓ | 9 |
| Anna Solé | 2018 | ✓ | ✓ | ✓ | ✓ | ✓ |  | ✓ | ✓ | ✓ | ✓ | 9 |
| Lei Han | 2019 | ✓ | ✓ | ✓ | ✓ | ✓ | ✓ | ✓ | ✓ | ✓ | ✓ | 10 |
| Amanda Ruth | 2021 | ✓ | ✓ | ✓ | ✓ | ✓ |  | ✓ | ✓ | ✓ | ✓ | 10 |
| Jennifer K  Workman | 2020 | ✓ | ✓ | ✓ |  | ✓ | ✓ | ✓ | ✓ | ✓ | ✓ | 9 |

Q1: Is the purpose of the research clear and the argument basis sufficient?

Q2: Is the selection of the study population clear?

Q3: Are the inclusion and exclusion criteria clearly described?

Q4: Are the sample characteristics clearly described?

Q5: Are the data collection tools reliable and valid?

Q6: Is it appropriate to verify the authenticity of the information of the study?

Q7: Are ethical issues taken into account?

Q8: Is the statistical method correct?

Q9: Are the presentation and analysis of the research results appropriate and accurate?

Q10: Is the value of research clearly stated?
